# Beyond the Scope: Unmasking Appendicitis Following Colonoscopy

**DOI:** 10.7759/cureus.106922

**Published:** 2026-04-12

**Authors:** Alaa Hajjar, Hind Joumaa, Fatima Alsalman, Mohamad El Haress, Zeena Haress, Walid Nasreddine

**Affiliations:** 1 Gastroenterology and Hepatology Department, Makassed General Hospital, Beirut Arab University, Beirut, LBN; 2 Gastroenterology Department, Makassed General Hospital, Beirut, LBN; 3 Department of Surgery, American University of Beirut Medical Center, Beirut, LBN; 4 Neurosciences, British Neuroscience Association, University of Sussex, Brighton, GBR

**Keywords:** appendicitis, bowel preparation, case report, colonoscopy complications, fecalith, open appendectomy, post-colonoscopy appendicitis

## Abstract

Colonoscopy is a key diagnostic and therapeutic procedure in gastrointestinal practice, although rare complications such as post-colonoscopy appendicitis (PCA) may occur. We report the case of a 61-year-old woman who presented 24 hours after a screening colonoscopy with severe diffuse abdominal pain, fever, nausea, vomiting, and watery diarrhea. The procedure had been complicated by poor bowel preparation, with a Boston Bowel Preparation Score of 1-2-1 (right colon, transverse colon, and left colon, respectively; scores range from 0 to 3 per segment, with 0 indicating an unprepared colon and 3 indicating perfectly clean mucosa). Abdominal computed tomography (CT) revealed phlegmonous acute appendicitis with abscess formation and a calcified fecalith, in a pelvis-projecting appendix with a medial cecal position. An open appendectomy was performed, given the complex intraoperative findings and unfavorable anatomy for laparoscopic access. The patient recovered uneventfully and was discharged the following day. This case highlights the convergence of poor bowel preparation, fecalith impaction, and complicated appendicitis as a mechanistically informative triad and underscores the importance of maintaining a low threshold for CT imaging in patients with new abdominal symptoms following colonoscopy.

## Introduction

Colonoscopy is among the most widely performed procedures in gastroenterology, serving both diagnostic and therapeutic purposes. In the United States alone, approximately 15 million colonoscopies are performed annually, with applications ranging from colorectal cancer screening to the management of inflammatory and neoplastic conditions [1,2]. The procedure carries a generally favorable safety profile; however, serious complications occur in 0.1-0.3% of cases, with higher rates following therapeutic interventions such as polypectomy and endoscopic mucosal resection [3]. While perforation and bleeding are the most recognized adverse events, rarer complications, including post-colonoscopy appendicitis (PCA), remain underrecognized due to their low incidence and overlapping presentation with other post-procedural sequelae [4].

PCA is estimated to occur in approximately 0.038% of procedures [4,5,6], although this figure likely underestimates the true incidence given the diagnostic challenges involved and inconsistencies in reporting. Epidemiological data suggest that the risk of developing appendicitis is significantly elevated in the week immediately following colonoscopy [6]. When further complicated by abscess formation, PCA presents additional management challenges and carries increased morbidity compared to uncomplicated cases.

This report describes a case of PCA with abscess formation in a 61-year-old woman following a screening colonoscopy with poor bowel preparation. Notably, the CT demonstrated a calcified fecalith and a pelvic-projecting appendix with a medial cecal position, a convergence of findings that directly informed both the pathogenic mechanism and the operative strategy. This case is of particular interest given the concurrence of poor bowel preparation, fecalith impaction, and complicated appendicitis as a mechanistically coherent triad that supports the luminal obstruction theory of PCA pathogenesis, and that has been rarely documented together in the existing literature [7,8]. By examining these features in depth, this report aims to contribute meaningful insights into the diagnosis, operative decision-making, and prevention of this underrecognized complication.

## Case presentation

A 61-year-old woman with a background of hypertension, on ramipril, and a one-pack-year smoking history presented to the emergency department with severe abdominal pain beginning twenty-four hours after a screening colonoscopy. She had no prior surgical history. Her ramipril was withheld on the morning of surgery in accordance with standard perioperative protocols, given the recognized risk of refractory intraoperative hypotension associated with angiotensin-converting-enzyme (ACE) inhibitor use.

The colonoscopy performed the previous day had been conducted under conscious sedation with midazolam and fentanyl. The procedure was well tolerated and without immediate complications; however, bowel preparation was inadequate, with a Boston Bowel Preparation Score of 1 (right colon), 2 (transverse colon), and 1 (left colon), indicating poor mucosal visualization across all segments [9]. Findings were limited to small internal hemorrhoids; the sigmoid, descending, transverse, and ascending colon, as well as the terminal ileum, were otherwise unremarkable. No evidence of cecal or appendiceal inflammation was noted endoscopically.

The pain was acute in onset, diffuse, non-radiating, and only partially relieved by paracetamol and nonsteroidal anti-inflammatory medications. Associated symptoms included fever reaching 38.5°C, nausea, and three episodes of vomiting. The vomiting was most likely attributable to the acute inflammatory process, given its onset twenty-four hours after the procedure, by which time the effects of conscious sedation with midazolam and fentanyl would be expected to have fully resolved; a residual post-sedation contribution cannot be entirely excluded, though the temporal course and concurrent fever argue in favor of an appendicitis-driven etiology. The patient also reported watery diarrhea, which in this context is most plausibly attributed to the residual effects of the bowel preparation agent rather than to the appendicitis itself, as secretory diarrhea is a well-recognized sequela of osmotic preparation and typically persists for up to 24-48 hours post-procedure. There was no melena or hematochezia.

On examination, the patient was febrile and exhibited right lower quadrant tenderness on palpation. Bowel sounds were present, and there were no peritoneal signs on initial assessment. Digital rectal examination revealed no blood. Laboratory investigations demonstrated leukocytosis with marked neutrophilia and an elevated C-reactive protein, consistent with an acute inflammatory process (Table 1). Mild hyponatremia (sodium 135 mEq/L) and a low bicarbonate (18 mEq/L) were also noted, likely reflecting a combination of poor oral intake, osmotic bowel preparation effects, and the systemic inflammatory response; both normalized with intravenous fluid resuscitation. 

**Table 1:** Laboratory findings on admission.

Laboratory parameter	Result	Reference range
WBC (cells/µL)	15,000 ↑	4,500–11,000
Hemoglobin (g/dL)	12	12.0–16.0
Neutrophils (%)	85 ↑	50–70
CRP (mg/L)	100 ↑	< 10
Creatinine (mg/dL)	0.8	0.6–1.1
Sodium (mEq/L)	135 ↓	136–145
Potassium (mEq/L)	3.6	3.5–5.0
Bicarbonate (mEq/L)	18 ↓	22–29

**Figure 1:**
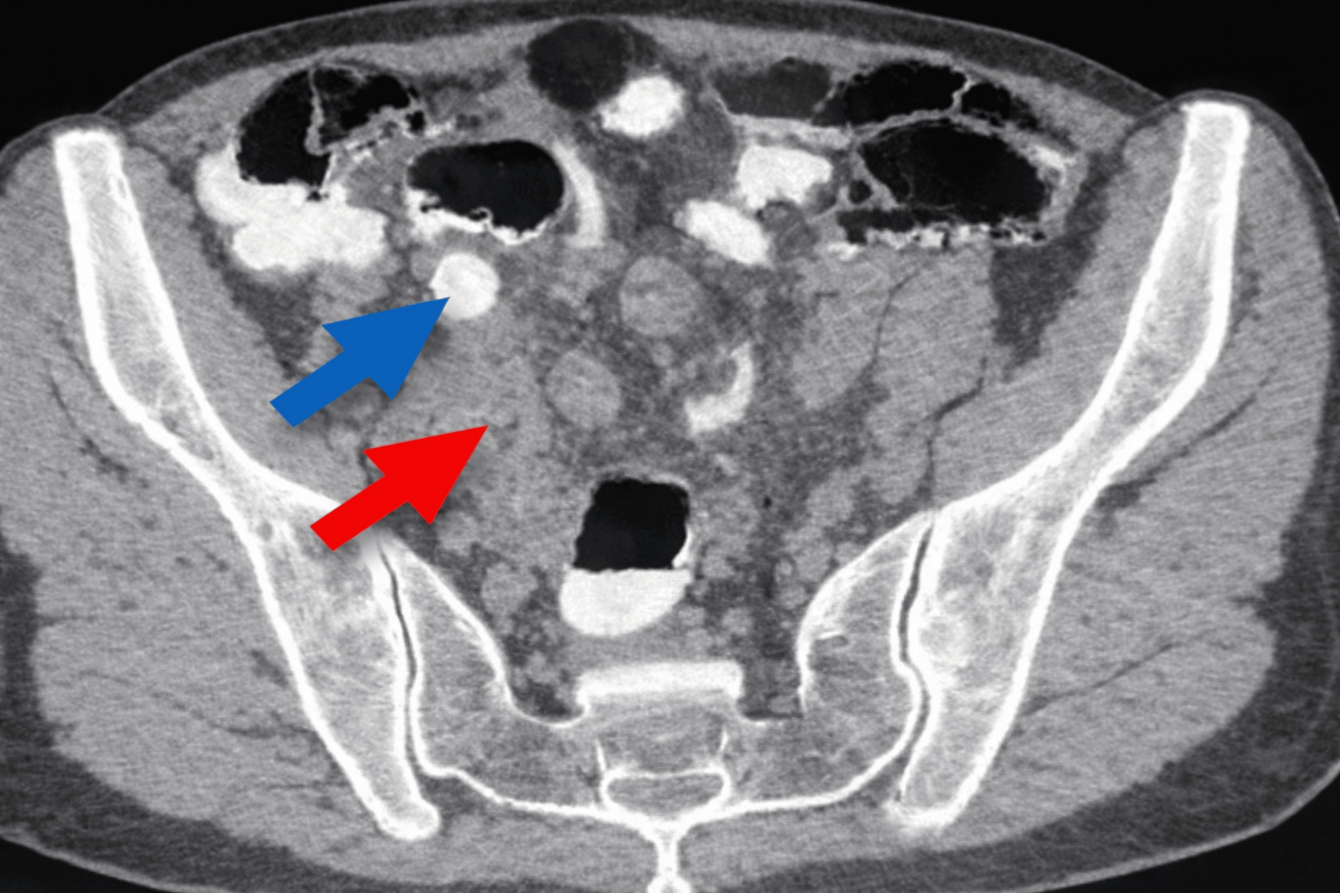
The appendix (red arrow), located medially to a right lower paramedian cecum with pelvic projection, is distended with a thick enhancing wall and a proximal calcified fecalith (blue arrow), with surrounding mesenteric fat stranding, findings consistent with phlegmonous acute appendicitis with abscess formation.

Contrast-enhanced computed tomography of the abdomen and pelvis revealed an appendix with medial positioning relative to a right lower paramedian cecum and pelvic projection. The appendix was distended with a thick, enhancing wall, a proximal calcified fecalith, and surrounding mesenteric fat stranding, consistent with phlegmonous acute appendicitis with abscess formation (Figure 1). 

A diagnosis of PCA was established. Intravenous fluid resuscitation was initiated, and broad-spectrum intravenous antibiotics (ceftriaxone 2 g) were administered approximately two hours prior to the surgical incision, in keeping with standard perioperative prophylaxis protocols. Informed consent was obtained from the patient prior to the procedure, with risks including conversion to open surgery, bleeding, infection, and visceral injury discussed. Following multidisciplinary surgical discussion, the decision was made to proceed with an open appendectomy. The rationale for an open rather than laparoscopic approach was based on: the CT evidence of dense periappendiceal phlegmon with abscess formation, which substantially increases the risk of inadvertent visceral injury under laparoscopic conditions; the pelvic projection and medial cecal position of the appendix, which created unfavorable ergonomics for laparoscopic dissection; and the high documented laparoscopic conversion rate in complicated PCA, reported at 19.2% in the largest systematic review to date [10,11]. An open approach was therefore selected to optimize operative safety and allow for thorough peritoneal lavage.

The open appendectomy was completed without complications, with a total operative time of 75 minutes. Postoperatively, the patient received intravenous antibiotics (ceftriaxone 2 g) and demonstrated an uneventful recovery. Renal function was monitored postoperatively, given the preoperative ACE inhibitor use; creatinine remained stable at 0.7 mg/dL. She was discharged the following day in stable condition with a prescription for oral ciprofloxacin 500 mg twice daily for seven days. Histopathological examination of the surgical specimen confirmed acute gangrenous appendicitis with periappendicitis. At one-week follow-up after discharge, the patient remained clinically well with no reported complications and appropriate wound healing.

## Discussion

Our patient's presentation fulfils all criteria proposed by Shaw, Gallardo, and Basson for PCA: appendicitis confirmed by standard diagnostic criteria, with symptom onset within 72 hours of colonoscopy, in the absence of endoscopic evidence of appendicitis or cecal inflammation at the time of the procedure [12]. PCA was first described by Houghton and Ashton in 1988 and remains a rare but clinically significant complication, with an estimated incidence of approximately 0.038%, or roughly 3.8 cases per 10,000 colonoscopies [10]. Basson and colleagues demonstrated that the risk of developing appendicitis is significantly elevated in the first week following colonoscopy compared to subsequent weeks, reinforcing the temporal relationship between the procedure and the complication [6].

The pathogenesis of PCA is not fully elucidated, but the prevailing theory centers on obstruction of the appendiceal lumen followed by progressive inflammation and bacterial overgrowth [10]. Several mechanisms have been proposed, including barotrauma from excessive insufflation, fecalith impaction into the appendiceal orifice, direct mucosal injury at or near the appendiceal opening, and exacerbation of pre-existing subclinical appendiceal pathology [13,14]. In our patient, the CT identification of a calcified fecalith at the proximal appendix is of particular mechanistic relevance. The poor bowel preparation, with a BBPS of 1-2-1, likely resulted in a high intraluminal fecal burden during the procedure. The combination of excessive insufflation pressure and residual fecal material may have driven the pre-existing fecalith more deeply into the appendiceal lumen, triggering complete obstruction and initiating the cascade of inflammation and abscess formation observed. This fecalith-driven, prep-facilitated mechanism is consistent with that described by Hamid et al. in their review of 57 PCA cases, where fecalith presence was documented in 31.3% of cases and poor bowel preparation was identified as a contributing factor in several instances [10].

In terms of patient demographics, our patient is a 61-year-old woman, which is somewhat atypical for PCA. Hamid et al. reported a median age of 55 years and a male predominance of 64.2% across published cases [10]. Female sex and older age may have contributed to the atypical initial presentation, with the absence of classical peritoneal signs on initial examination despite the presence of abscess formation on imaging, a discrepancy that underscores the importance of CT in this clinical context. Although hypertension and a remote smoking history were present, neither has been established as an independent risk factor for PCA or for surgical complications in appendicitis; these comorbidities were nonetheless optimally managed perioperatively, including withholding of the ACE inhibitor prior to surgery. The symptom onset at 24 hours is consistent with the most common presentation window reported in the literature, with 79.1% of PCA patients developing symptoms within 48 hours of the procedure [10].

PCA poses a diagnostic challenge, as its presentation overlaps substantially with other post-procedural complications, including bowel perforation, post-polypectomy syndrome, and mesenteric ischemia [15,16,17]. In our patient, the absence of free peritoneal air on CT effectively excluded perforation, and the absence of mucosal changes on colonoscopy argued against post-polypectomy syndrome. Contrast-enhanced CT was pivotal in establishing the diagnosis, identifying the appendiceal inflammation, abscess, and fecalith with high specificity. This is consistent with the current evidence base: CT has become the predominant diagnostic modality in PCA, used in over 70% of reported cases, while ultrasound and plain radiography offer limited sensitivity and specificity in this setting [16]. Biochemically, our patient presented with leukocytosis and marked neutrophilia (WBC 15,000 cells/µL, neutrophils 85%) alongside a significantly elevated CRP (100 mg/L), a pattern consistent with complicated rather than uncomplicated appendicitis and correlating with the CT findings of abscess formation.

The management of PCA mirrors that of standard acute appendicitis and is guided by the degree of complexity at presentation. For uncomplicated cases, non-operative management with intravenous antibiotics alone has shown feasibility, with Ng et al. reporting successful antibiotic treatment in four of twelve conservatively managed cases (33.3%) in their systematic review [7]; however, this approach is not appropriate in the context of abscess formation, as was present in our patient. Among those requiring surgery, the largest systematic review of PCA to date, comprising 67 patients, found that open appendectomy was performed in 56.8% of cases, with a laparoscopic conversion rate of 19.2% and complicated appendicitis (perforation or abscess) present in 49.2% of surgical cases [11]. The high rate of operative complexity in PCA likely reflects the dense periappendiceal inflammation that characterizes procedure-related appendicitis, in which the tissue planes are often more hostile than in spontaneous appendicitis. In our patient, the decision to proceed with a primary open approach was further supported by the pelvic projection and medial cecal position of the appendix, which would have created significant ergonomic challenges for laparoscopic dissection. The open approach permitted direct tactile assessment, safe dissection of the phlegmonous tissue, and thorough peritoneal lavage [11,18,19]. The patient received postoperative intravenous antibiotics and was discharged the following day with an uncomplicated recovery.

The discrepancy between the CT diagnosis of phlegmonous appendicitis and the histopathological finding of gangrenous appendicitis warrants discussion. CT findings of phlegmonous appendicitis, characterized by mural thickening, periappendiceal fat stranding, and abscess formation without frank perforation, represent a radiological snapshot at the time of imaging, which in this case preceded surgery by several hours. Gangrenous appendicitis reflects transmural necrosis that may not yet be radiologically apparent in its early stages; CT has well-recognized limitations in distinguishing phlegmonous from early gangrenous disease, with reported sensitivity for complicated appendicitis ranging from 64% to 88% and overall accuracy of 81% on conventional contrast-enhanced imaging [20], and with up to 22% of patients found intraoperatively to have more advanced disease than CT suggested [21]. It is therefore likely that gangrenous progression had either already occurred beyond what CT could reliably detect at the time of imaging or that the disease advanced to transmural necrosis in the interval between imaging and surgical intervention, a recognized phenomenon in complicated appendicitis. This underscores the importance of intraoperative assessment as the definitive arbiter of disease severity and reinforces the appropriateness of the open approach selected in this case.

The clinical value of this case lies in the convergence of three factors that together define a high-risk PCA phenotype: grossly inadequate bowel preparation, CT-confirmed fecalith impaction, and complicated appendicitis with abscess formation requiring open surgery. While each of these features has been individually documented in PCA reports, their concurrence in a single case with clear mechanistic coherence, linking poor prep to fecalith displacement and luminal obstruction, is infrequently reported and adds to the pathophysiological understanding of this entity [7,8]. Furthermore, the anatomical variant of a pelvic-projecting appendix with medial cecal positioning contributed both to the risk of PCA and to operative complexity, a combination that highlights the role of appendiceal anatomy in modulating clinical course. Clinicians performing colonoscopy should be aware that poor bowel preparation, particularly in patients with known or suspected fecalith, may represent a modifiable risk factor for PCA, warranting consideration of repeat or extended preparation before the procedure.

Limitations

As with all single-institution case reports, certain inherent constraints apply. The absence of extended follow-up beyond one week reflects the patient’s uncomplicated early recovery and discharge; longer surveillance was not clinically indicated at the time. The precise intraoperative mechanism of fecalith displacement cannot be directly observed and is necessarily inferred from the convergence of CT findings and procedural context, an approach consistent with the inferential reasoning applied across the PCA literature. The role of poor bowel preparation as a precipitating factor, while mechanistically coherent, cannot be established as causal from a single case. These considerations notwithstanding, this report contributes a well-documented example of a high-risk PCA phenotype with clear pathophysiological reasoning and adds to the growing body of evidence informing the diagnosis and management of this underrecognized complication.

## Conclusions

PCA is a rare but clinically important complication that should be considered in any patient presenting with new or worsening abdominal symptoms following colonoscopy, regardless of the apparent success of the procedure. This case illustrates how the convergence of poor bowel preparation, fecalith impaction, and an anatomically unfavorable appendix can precipitate complicated PCA requiring open surgical intervention. CT imaging is the cornerstone of diagnosis and should be obtained promptly. A primary open approach is appropriate when CT demonstrates abscess formation or periappendiceal phlegmon, and clinicians should recognize that intraoperative findings may reveal more advanced disease, including gangrenous change, than CT alone can reliably predict. Clinicians and endoscopists alike should maintain heightened vigilance in patients with inadequate bowel preparation and be prepared to act decisively when PCA is suspected.
